# Relationship between preoperative neutrophil‐to‐lymphocyte ratio and postoperative delirium: The PNDABLE and the PNDRFAP cohort studies

**DOI:** 10.1002/brb3.3281

**Published:** 2023-10-13

**Authors:** Xiaoyue Wu, Feifei Chi, Bin Wang, Siyu Liu, Fei Wang, Jiahan Wang, Xinhui Tang, Yanlin Bi, Xu Lin, Jun Li

**Affiliations:** ^1^ Department of Anesthesiology Qingdao Hospital, University of Health and Rehabilitation Sciences (Qingdao Municipal Hospital) Qingdao China; ^2^ Department of Anesthesiology Qingdao Eighth People's Hospital Qingdao China; ^3^ Department of the Third Central Clinical College of Tianjin Medical University Tianjin China; ^4^ School of Anesthesiology Weifang Medical University Weifang China; ^5^ Department of Neurology Qingdao Hospital, University of Health and Rehabilitation Sciences (Qingdao Municipal Hospital) Qingdao China

**Keywords:** Alzheimer‐related biomarkers, cognitive, NLR, POD, risk factor

## Abstract

**Objectives:**

In this study, the relationship between preoperative neutrophil‐to‐lymphocyte ratio (NLR) and Alzheimer‐related biomarkers in cerebrospinal fluid (CSF) was investigated to determine whether high NLR is a potential risk factor for postoperative delirium (POD) and to evaluate its predictive efficacy.

**Methods:**

We selected 1000 patients from the perioperative neurocognitive disorder risk factor and prognosis (PNDRFAP) database and 999 patients from the perioperative neurocognitive disorder and biomarker lifestyle (PNDABLE) database. Patients in the PNDABLE database have been measured for Alzheimer‐related biomarkers in CSF (Aβ_40_, Aβ_42_, P‐tau, and tau protein). Mini‐mental state examination was used to assess the preoperative mental status of patients. POD was diagnosed using the confusion assessment method and assessed for severity using the memorial delirium assessment scale. Logistic regression analysis was utilized to explore the association of preoperative NLR with POD. What's more, we also performed sensitivity analysis by adding corrected confounders, and the results were almost unchanged. Spearman's rank correlation was used to determine the associations between NLR and Alzheimer‐related biomarkers. Mediation analyses with 10,000 bootstrapped iterations were used to explore the mediation effects. Finally, we use decision curves and the nomogram model to evaluate the efficacy of preoperative NLR in predicting POD; we also performed external validation using data from Qilu Hospital.

**Result:**

Logistic regression results showed that an elevated preoperative NLR was a risk factor for the development of POD in patients (PNDRFAP: OR = 1.067, 95% CI 1.020–1.116; PNDABLE: OR = 1.182, 95% CI 1.048–1.335, *p* < .05). Spearman's rank correlation analysis showed a positive but weak correlation between NLR and P‐tau/T‐tau (*R* = .065). The mediating effect results indicate that NLR likely mediates the occurrence of POD through elevated tau protein levels (proportion: 47.47%). The results of the box plots showed statistically significant NLR and CSF biomarkers between the POD and non‐POD (NPOD) groups (*p* < .05), with higher NLR, P‐tau, and T‐tau in the POD group than in the NPOD group. In contrast, the NPOD group had higher Aβ_42_ levels compared to the POD group. In addition, we used R package to plot the decision curve and nomogram both suggesting a good predictive effect of preoperative NLR on the occurrence of POD.

**Conclusion:**

Elevated preoperative NLR levels may be a risk factor for POD and likely mediate the development of POD through elevated P‐tau/T‐tau levels.

## Graphical abstract

This is the first prospective population‐based study to assess the relationship between the prevalence of POD and preoperative NLR levels. This study proposes that elevated preoperative NLR levels are a risk factor for POD, and elevated P‐tau and T‐tau are also associated with the development of POD. However, AÎ^2^42 is a protective factor for POD. Preoperative NLR has the potential to act as a predictor for the development of POD. We can reduce the occurrence of POD through possible interventions to bring down preoperative NLR levels.

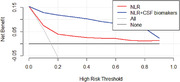

## 1 INTRODUCTION

Postoperative delirium (POD) can be defined as acute cerebral dysfunction or failure and fluctuating consciousness, accompanied by an obvious impairment of concentration and cognitive function (Rudolph & Marcantonio, 2011; Zenilman, 2017). It often occurs in the first week after surgery (or before discharge) with a higher incidence 1–3 days postoperatively (Evered et al., 2018). Previous studies have shown that the incidence of POD is about 17.6% (Rong et al., 2021). POD may lead to higher health resource costs and mortality (Glumac et al., 2019). However, studies related to the pathological mechanism of POD are not thorough nowadays, which prompts us to find an effective way to predict POD.

Previous clinical and animal studies have shown that many inflammatory cytokines can disrupt the blood–brain barrier (BBB) through the central nervous system, leading to neurocognitive dysfunction (Wen et al., 2020; Zheng et al., 2017). Extensive research suggests that inflammatory pathophysiology plays a key role in modulating cognitive impairment (Darweesh et al., 2018; Kuyumcu et al., 2012; Zha et al., 2021). Neutrophil‐to‐lymphocyte ratio (NLR) is a marker of inflammation and oxidative stress (He et al., 2020; Pohjasvaara et al., 2007). A study showed that NLR in peripheral blood can also be used as a simple systemic inflammatory response (SIR) marker and has diagnostic value for certain diseases (Celikbilek et al., 2013; He et al., 2020). NLR has been shown to be a good predictor of neurological and psychiatric disease outcomes (Kulaksizoglu & Kulaksizoglu, 2016; Liu et al., 2020; Xue et al., 2017).

Previous studies have shown that lower preoperative cerebrospinal fluid (CSF) Aβ levels and higher tau levels are associated with a higher incidence of POD and more severe symptoms (Idland et al., 2017; Querfurth & LaFerla, 2010). CSF Aβ_40_, Aβ_42_, T‐tau, and P‐tau have been reported in numerous studies to help predict cognitive dysfunction. It was also found that patients with POD had a lower Aβ_42_/P‐tau ratio and a lower CSF Aβ_42_ level (Hu et al., 2017; Idland et al., 2017; Xie et al., 2014).

NLR is calculated by dividing the absolute neutrophil count by the absolute lymphocyte count. Recently, NLR has become a hot topic of research in various diseases, such as cardiovascular diseases and cancer (Zha et al., 2021). However, so far, far less attention has been paid to the relationship between NLR and POD.

In order to reveal the relationship between them, this study analyzed from the following three perspectives. At first, whether NLR was an independent influence on POD. Second, is there a correlation between Alzheimer‐related biomarkers and NLR. Third, if a correlation does exist, whether NLR will lead to POD through Alzheimer‐related biomarkers. If the above conjecture was confirmed, the study would hopefully provide a preemptive strategy to decrease the incidence of POD in the elderly and reduce the burden it takes to families and the society.

## 2 METHODS

### 2.1 Study enrollment

Data for this study were obtained from voluntary participants in the perioperative neurocognitive disorder risk factor and prognosis (PNDRFAP) study (Deng et al., 2022) and the perioperative neurocognitive disorder and biomarker lifestyle (PNDABLE) study (Lin et al., 2021; Wang et al., 2021).

The PNDRFAP study (Deng et al., 2022) and the PNDABLE study (Lin et al., 2021; Wang et al., 2021), which are two ongoing large‐scale cohort studies initiated in 2020 that focus on risk factors and biomarkers of perioperative neurocognitive disorders (PND) in Han populations in northern China. The purpose of these two databases is to identify genetic and environmental factors of PND biomarkers and lifestyles that may modify the risk of PND in non‐demented Han populations in northern China, thus forming the basis for disease prevention and early diagnosis. All participants were given informed consent, and they have the right to withdraw at any time and for any reason. Their CSF and blood samples could be used for research purposes in the future.

In PNDRAFP study, the inclusion criteria included: (1) The patients were aged 40–90 years old; (2) American Society of Anesthesiologists physical status (ASA) I–III; (3) the patients had intact preoperative cognitive function without communication disorders; (4) the patients had sufficient education to complete the preoperative neuropsychological tests; (5) patients undergoing gastrointestinal/hepatobiliary surgery and genitourinary surgery under general anesthesia. Exclusion criteria included: (1) mini‐mental state examination (MMSE) scores are less than 24; (2) preoperative abnormal coagulation function, hematologic disorders; (3) serious psychological disorders or deaf patients.

In PNDABLE study, the inclusion criteria included: (1) the patients were aged 40–90 years old; (2) ASA I–III; (3) patients undergoing knee/hip replacement surgery under combined spinal–epidural anesthesia (CSEA); (4) the patients had intact preoperative cognitive function without communication disorders; (5) the patients had sufficient education to complete the preoperative neuropsychological tests. Exclusion criteria included: (1) preoperative MMSE score is less than 24; (2) drug or psychotropic substance abuse, as well as long‐term use of steroid drugs and hormone drugs; (3) hematologic disorders (e.g., leukocytosis and lymphocytic leukemia); (4) recent major surgery; (5) severe visual and hearing impairments; (6) abnormal coagulation function before surgery; (7) central nervous system infection, head trauma, multiple sclerosis, neurodegenerative diseases other than Alzheimer's disease (AD), or other major neurological disorders; (8) major psychological disorders; (9) severe systemic diseases that may affect CSF or blood levels of Alzheimer‐related biomarkers including Aβ and tau protein; (10) family history of genetic diseases (e.g., early‐onset familial AD, hereditary ataxia, and hereditary spastic paraplegia).

### 2.2 Sample size estimation

The preliminary test in this study explored that eight covariates (NLR, Aβ_40_, Aβ_42_, P‐tau, T‐tau, age, male, MMSE) were included in the logistic regression. According to preliminary test, the POD incidence was 9.8%, and the loss of follow‐up rate was assumed to be 20%. Thus, the required sample size was calculated to be 1021 cases (8 × 10 ÷ 0.098 ÷ 0.8 = 1021).

### 2.3 Anesthesia and surgery

All patients were prohibited from drinking for 6 h and eating for 8 h prior to surgery. Upon entering the operating room, we routinely monitored ECG, SpO2, and NBP and opened venous access.

Patients in the PNDRFAP study were anesthetized by general anesthesia. Anesthesia was induced by sufentanil 0.2–0.5 μg/kg, ceftriaxonium 0.15–0.2 mg/kg, and etomidate 0.15–0.3 mg/kg. Intraoperative analgesia was maintained by the continuous pumping of remifentanil 0.25–2 μg/kg min. Depending on the depth of anesthesia, 0.5%–3% sevoflurane was inhaled.

Patients in the PNDABLE study received combined spinal and epidural anesthesia. The anesthesia position was lateral, and the space between the lumbar 3 and 4 (L3 and L4) vertebrae was used as the puncture site. After successful puncture, 2 mL of CSF was aspirated from the subarachnoid space, and then, 2–2.5 mL of ropivacaine (0.66%) was injected within 30 s. CSF samples were centrifuged at 2000 *g* for 10 min, and the CSF supernatant was stored in enzyme‐free EP (Eppendorf) tubes (AXYGEN; PCR‐02‐C) at −80°C for subsequent studies. During the procedure, the patient's vital signs were maintained stable by administering appropriate vasoactive drugs.

After surgery, the patient was routinely admitted to the anesthesia recovery room and observed for 30 min. The numerical rating scale (NRS) was used to assess the pain. Patient‐controlled intravenous analgesia was used for pain relief (butorphanol tartrate injection 10 mg + toranisetron hydrochloride injection 5 mg + 0.9% sodium chloride solution 89 mL maintained NRS < 3 points).

### 2.4 Data collection

Blood samples are drawn the day before surgery from 8:00 to 10:00 a.m. and routinely sent to the laboratory for preoperative testing, and the postoperative statistical results are logged into the database. Baseline data and living habits of patients are obtained through electronic medical records and preoperative visits, which are unified and logged into the database as well.

### 2.5 Neuropsychological testing

All patients accepted meticulous clinical and neuropsychological assessments and MMSE the day before the scheduled operation. Patients were followed up on postoperative days 1–7 or before they were discharged from the hospital at 10 a.m. and 2 p.m. twice a day. At the same time, the presence or absence of POD was recorded. The presence of POD was defined according to the confusion assessment method (CAM), those with POD were classified as POD group and those with POD negative were classified as non‐POD (NPOD) group. The severity of POD was defined according to the memorial delirium assessment scale (MDAS) (Inouye et al., 1990; Schuurmans et al., 2003). All of the above assessments were performed by an anesthesiologist and a neurologist who had no knowledge of the patient's perioperative management (The anesthetist and neurologist who visit preoperatively and postoperatively are different). The CAM and MDAS have been proven to apply to the patients with good credibility and practicality (Leung et al., 2008; Shi et al., 2014).

### 2.6 Measurements of CSF sampling

A volume of 2 mL CSF was collected in a polypropylene centrifugal tube, then centrifuged at 2000 × *g* for 10 min at room temperature (Bakr et al., 2017; Pérez‐Ruiz et al., 2018) as well as separated and stored in an enzyme‐free EP (Eppendorf) tube (oxygen bottle, PCR‐02‐C) at −80°C for further use in the following steps of this study. These samples were subjected to at most two freeze–thaw cycles.

ELISA was used to detect the levels of Aβ_40_, Aβ_42_, T‐tau, and P‐tau, which were detected from 2 mL CSF, using Aβ_40_ (BioVendor, Lot: No. 292‐6230), Aβ_42_ (BioVendor, Lot: No. 296‐64401), P‐tau (BioVendor, Lot: QY‐PF9092), and tau (BioVendor, Lot: No. EK‐H12242) assay kit under the manufacturer's instructions. Finally, using an enzyme marker (EnSpire, PerkinElmer) (Bakr et al., 2017; Pérez‐Ruiz et al., 2018) to measure the optical density value (OD value) of each hole at the wavelength of 450 m. All samples were measured by the same laboratory personnel, and they were blinded with the group assignment.

### 2.7 Statistical analysis

The Kolmogorov–Smirnov test was used to determine the normality of the sample. Data conforming to a normal distribution were expressed as mean ± standard deviation, and data not conforming to a normal distribution were expressed as median (interquartile spacing) [M(Q)] or as a number (%). A *t*‐test of two independent samples was used to test whether there were differences in CSF biomarker levels and NLR between the POD and NPOD groups.

Binary logistic regression was used to discuss whether NLR was a risk factor for POD and the effect of the four CSF biomarkers on POD. To further verify the accuracy of the results and exclude the influence of confounding factors, three models were developed for sensitivity analysis: (1) Three confounding factors of gender, age, and MMSE were included; (2) confounding factors of hypertension, diabetes, coronary heart disease, and years of education were further included on the basis of (1); and (3) patients aged ≥65 years were further analyzed.

Spearman's rank correlation was used to determine the associations between NLR and Alzheimer‐related biomarkers.

In addition, we performed a mediating effect to examine the relationship among NLR, POD, and CSF biomarkers. A mediating effect would be established if the following criteria were met simultaneously: (1) Changes in NLR significantly affected Alzheimer's‐related biomarkers in CSF; (2) changes in Alzheimer's‐related biomarkers in CSF were responsible for changes in POD; (3) changes in NLR were significantly or insignificantly related to POD; (4) the inclusion of Alzheimer's‐related biomarkers in the regression model–related biomarkers, the relationship between NLR and POD was attenuated. In addition, attenuation or indirect effects were estimated, using 10,000 bootstrap iterations to determine significance, where each path of the model controlled for Age, Gender, and MMSE.

The predictive values of NLR and CSF biomarkers are described using decision‐making. Nomogram will be used to visualize the prediction results and calibration curves will be used to validate the predicted models. We also used the DynNom package to create a Dynamic nomogram (https://thedile.shinyapps.io/dynnomapp/) to predict the likelihood of POD.

The difference was considered statistically significant at *p* < .05.

The data were analyzed with R4.4.1 (R Foundation for Statistical Computing) and Stata MP16.0 (Solvusoft Corporation, Inc).

## 3 RESULTS

### 3.1 Characteristics of participants

Finally, 1000 (PNDRFAP) and 999 (PNDABLE) patients were included in this study by statistically organizing the database and excluding missing data, lost visits, and surgical cancellations (see the flow diagram).

In the PNDRFAP cohort, a total of 1000 patients were included, and 154 subjects developed POD at the 1–7‐day postoperative follow‐up (positivity rate for POD: 15.4%). We found no differences in age and MMSE between the POD and NPOD groups, except for higher levels of NLR in the POD group compared to the NPOD group.

For the PNDABLE cohort, which included a total of 999 patients, the incidence of the same POD was 15.4%. In addition to age, MMSE differed between the two groups, NLR was higher in the POD group than in the NPOD group, and Aβ_42_, T‐tau, and P‐tau differed between the POD and NPOD groups.

Specific information for all participants is shown in Table 1.

### 3.2 Comparison between POD and NPOD groups

**FIGURE 1:**
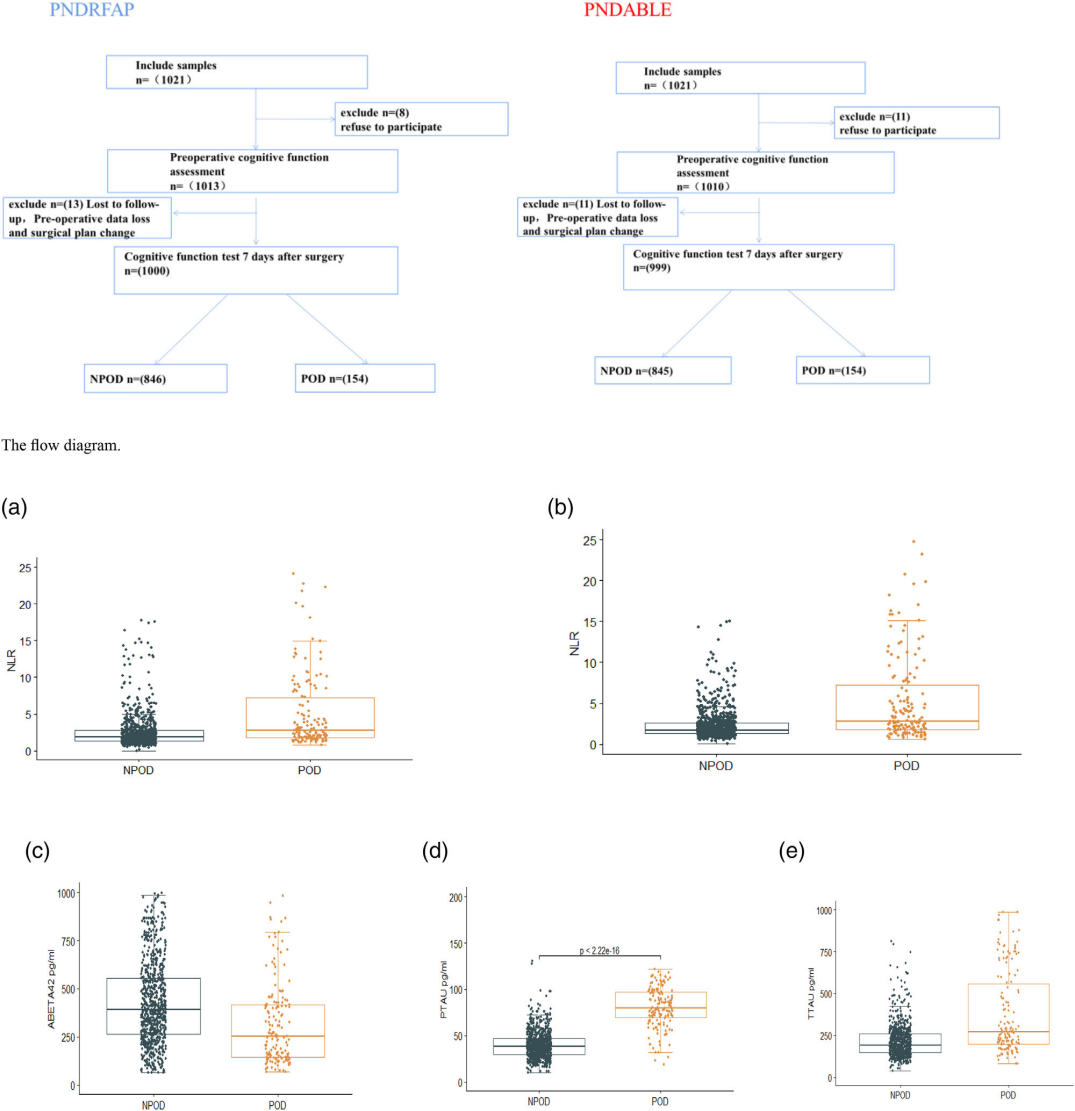
Expression of cerebrospinal fluid (CSF) biomarkers and neutrophil‐to‐lymphocyte ratio (NLR) of postoperative delirium (POD) patients and non‐POD (NPOD) controls. The scatter plots showed the expression levels of NLR ((A) PNDRFAP and (B) PNDABLE) and Aβ_42_ (C), P‐tau (D), T‐tau (E). The colors of scatter maps are grouped according to different diagnostic groups. The *p* value was determined by the two independent samples *t*‐test. The levels of CSF Aβ_42_, P‐tau, T‐tau, and NLR in patients with POD group were significantly different than those in patients NPOD group (*p* < .001).

Participants were divided into two groups (POD and NPOD) according to the occurrence of POD, and two independent samples *t*‐tests were performed in order to compare the levels of CSF biomarkers and NLR profiles. The results showed a significant difference in NLR, Aβ_42_, P‐tau, and T‐tau between the POD and NPOD groups (*p* < .05). Both databases showed higher levels of NLR in the POD group. P‐tau and T‐tau were higher in the POD group than in the NPOD group in the PNDABLE database, whereas the NPOD group had more Aβ_42_. Please refer to Figure 1 for the study results.

### 3.3 Results of binary logistic regression and sensitivity analysis

Binary logistic regression results yielded that both the PNDRFAP database (OR = 1.158, 95% CI 1.107–1.212, *p* < .05) and the PNDABLE database (OR = 1.208, 95% CI 1.122–1.301, *p* < .05) showed high levels of preoperative NLR as a risk factor for POD. The relationship between CSF biomarkers and POD could also be inferred in the PNDABLE database; people with higher Aβ_42_ levels may have a lower risk of POD (OR = .998, 95% CI .996–.999, *p* < .05); P‐tau (OR = 1.099, 95% CI 1.082–1.116, *p* < .05), and T‐tau (OR = 1.003, 95% CI 1.001–1.004, *p* < .05) as risk factors for POD.

In order to minimize the influence of confounding factors and to improve the reliability of the results, a sensitivity analysis was performed: This was done by building three models, (1) three confounding factors of gender, age, and MMSE were included; (2) confounding factors of hypertension, diabetes, coronary heart disease, and years of education were further included on the basis of (1); and (3) patients aged ≥65 years were further analyzed. By sensitivity analysis, we reached consistent conclusions (PNDRFAP: OR = 1.067, 95% CI 1.020–1.116; PNDABLE: OR = 1.182, 95% CI 1.048–1.335, *p* < .05) (please refer to Tables 2 and 3 for the study results).

### 3.4 Analysis of Spearman's rank correlation

**FIGURE 2:**
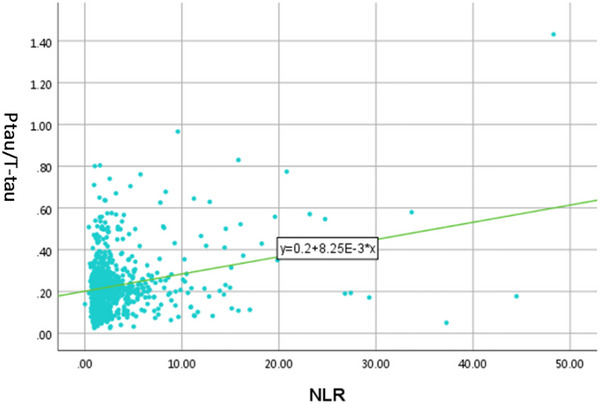
Associations of neutrophil‐to‐lymphocyte ratio (NLR) and P‐tau/T‐tau. Scatter plots represent the associations of NLR with P‐tau/T‐tau (*R* = .065, *p* = .045).

In logistic regression analysis, both T‐tau and P‐tau were independent risk factors for POD, so we used P‐tau/T‐tau as a predictor of POD. Spearman's rank correlation looked at the relationship between NLR and P‐tau/T‐tau. Spearman's rank correlation analysis showed a positive but weak correlation between NLR and P‐tau/T‐tau (*R* = .065). Please refer to Figure 2 for the study results.

### 3.5 Analysis of mediating effects

**FIGURE 3:**
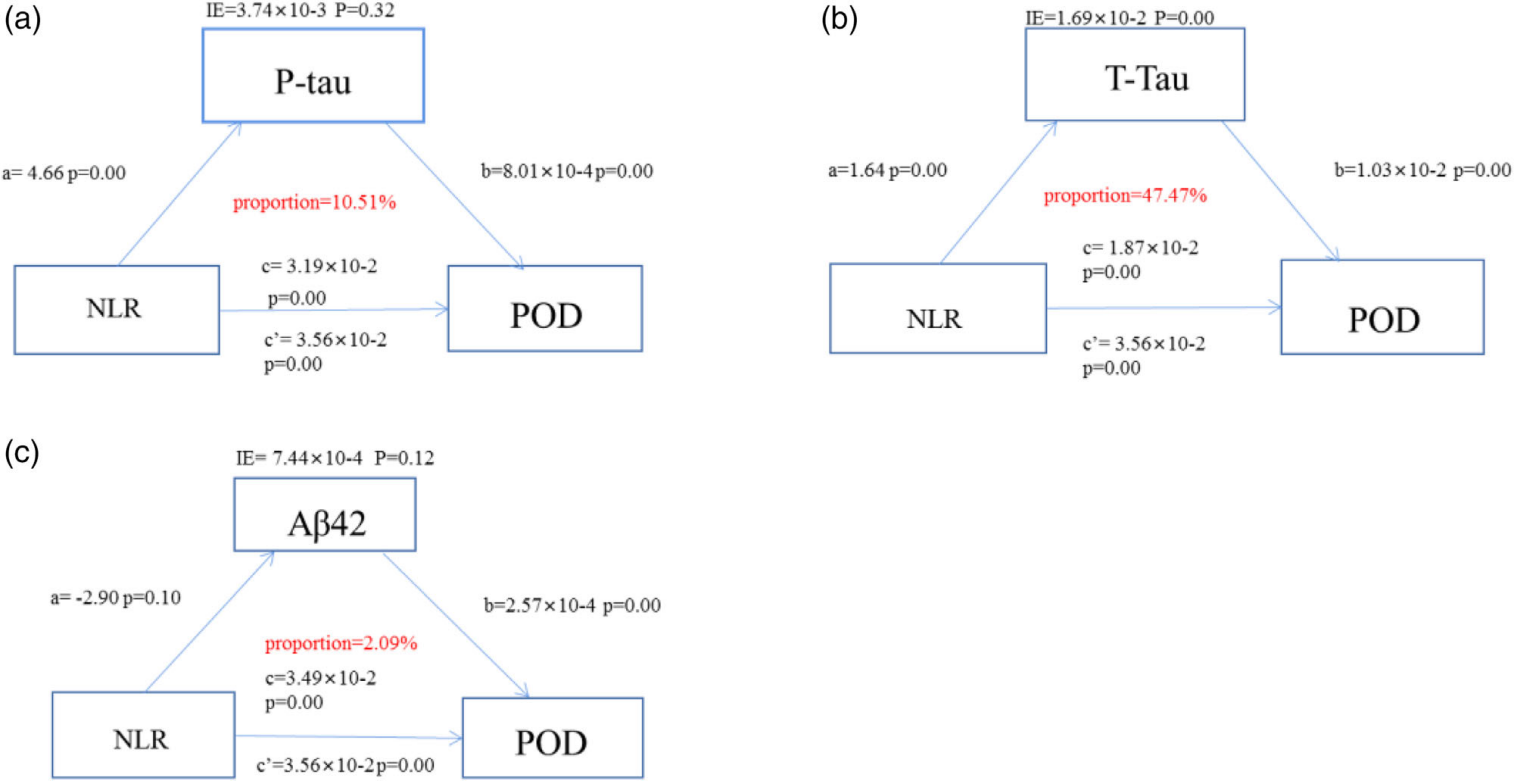
Mediation analyses with 10,000 bootstrapped iterations were used to examine mediation effects of (A) P‐tau, (B) T‐tau, (C) Aβ_42_ on postoperative delirium (POD). Relationship between neutrophil‐to‐lymphocyte ratio (NLR) and POD was mediated by cerebrospinal fluid (CSF) T‐tau (proportion = 47.47%), P‐tau (proportion = 10.51%), Aβ_42_ (proportion = 2.09%). Aβ_42_, β‐amyloid42; IE, indirect effect; P‐tau, phosphorylated tau; T‐tau, total tau.

In the above results, it is not difficult to find that NLR is a risk factor for POD; Alzheimer's disease‐related biomarkers are also associated with the occurrence of POD. What is more, there is a positive correlation between P‐tau/T‐tau and NLR. To investigate what kind of relationship exists among the three, we conducted mediating effects. We found that the relationship between NLR and POD was mediated by CSF P‐tau, T‐tau (proportion: 10.51%; proportion: 47.47%), and Aβ_42_ (proportion: 2.09%). The percentage of Aβ_42_ < 10%, so it does not play a significant role (Figure 3).

### 3.6 Predictive model

**FIGURE 4:**
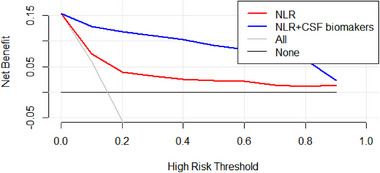
Decision curve.

**FIGURE 5:**
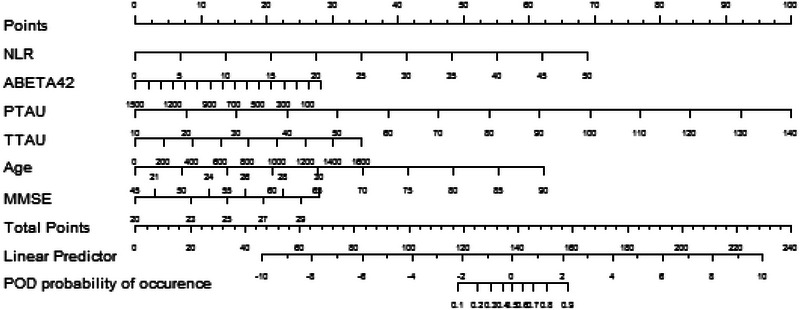
Neutrophil‐to‐lymphocyte ratio (NLR), P‐tau, T‐tau are risk factors for postoperative delirium (POD), and within a certain range, the risk of developing POD increases as NLR, P‐tau, T‐tau; Aβ_42_ is a protective factor for POD, and the risk of developing POD increases as the level of Aβ_42_ decreases.

**FIGURE 6:**
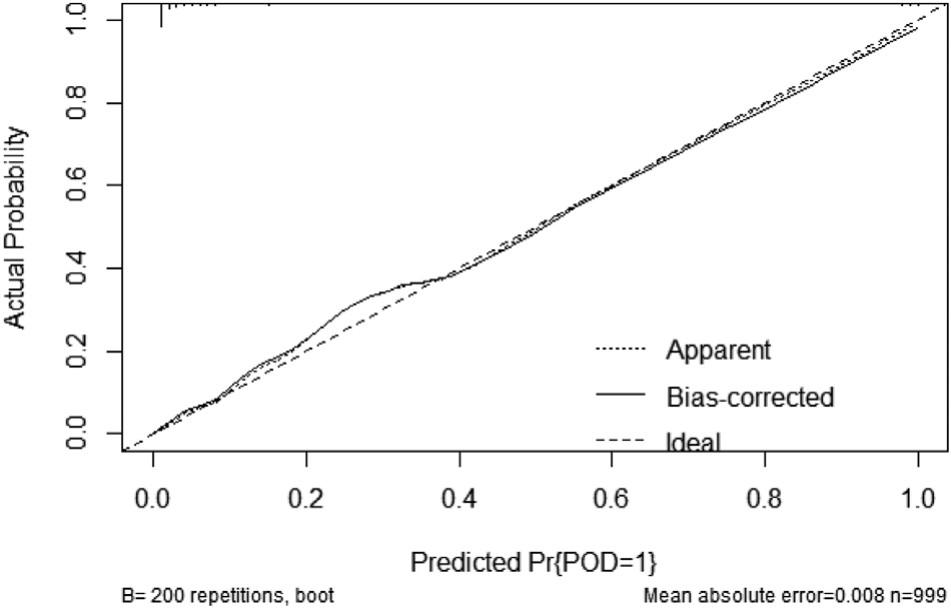
The calibration curve indicated good prediction of the nomogram.

The results of the decision curve suggest that NLR (Red Line) has a good predictive effect on POD, and the results of this study show that if Alzheimer's disease‐related biomarkers + NLR (Blue Line) is used as a preoperative predictor at the same time, the prediction of POD can be more accurate (Figure 4). The efficacy of each predictor is shown in the nomogram (Figure 5). The calibration curve indicated a good prediction of the nomogram (Figure 6).

**FIGURE 7:**
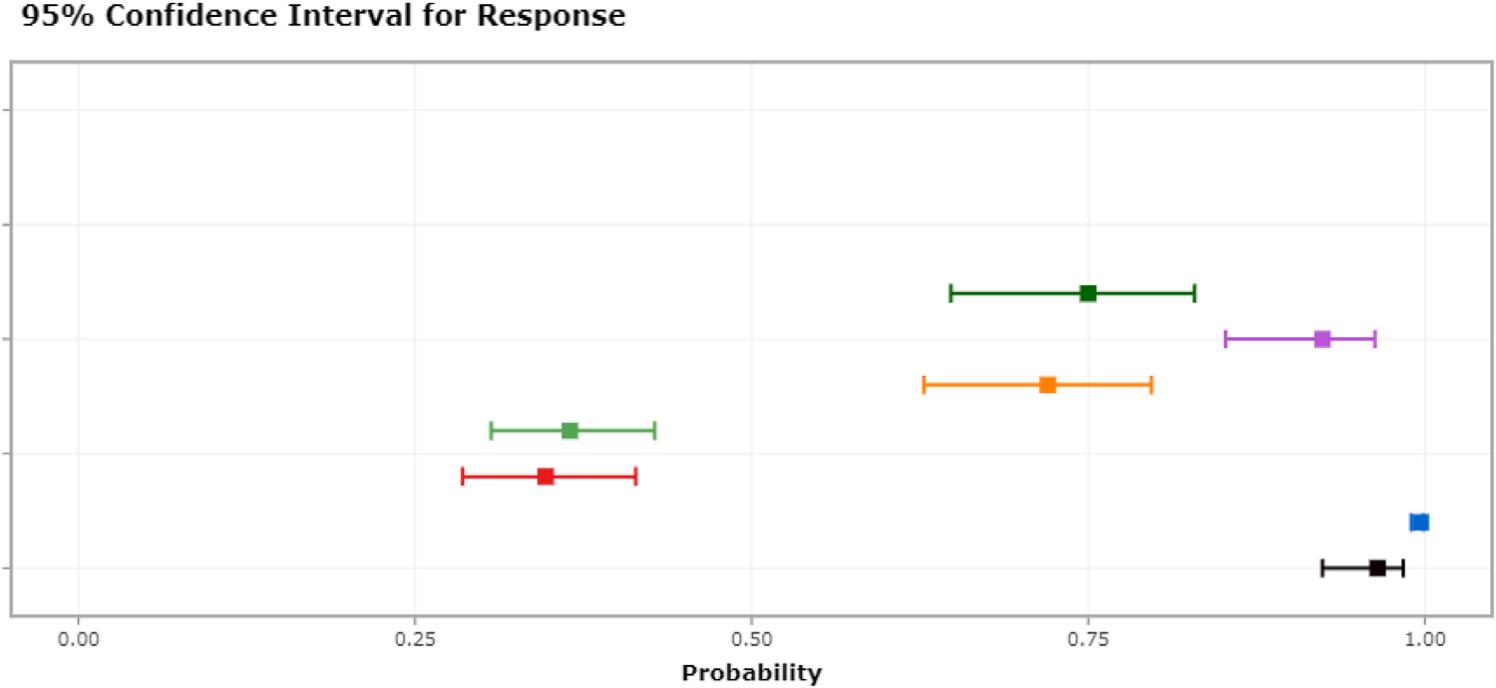
Dynamic nomogram (data for external validation were obtained from Qilu University School of Medicine; https://thedile.shinyapps.io/dynnomapp/).

Our external validation using data from Qilu Hospital showed that the higher the preoperative NLR level and the older the age, the greater the risk of developing POD (Figure 7).

## 4 DISCUSSION

In this study, we investigated the relationship among preoperative NLR, Alzheimer‐related biomarkers, and POD. The results of this study suggest that elevated preoperative NLR level may be a risk factor for POD (Weston et al., 2017) and is associated with elevated TAU protein level. Among them, NLR influences the occurrence of POD through T‐tau and P‐tau. This is similar to the findings of Sayed et al. (2020).

Some researchers have suggested that the risk of cognitive dysfunction can be reduced by early intervention (Dong et al., 2019). Moreover, it has long been demonstrated that inflammation is associated with Tau protein phosphorylation (Quintanilla et al., 2004) and NLR is a marker of peripheral inflammation. Therefore, it is urgent to actively explore the relationship among NLR, POD, and CSF biomarkers to reduce the incidence of POD.

POD is characterized by acute impairment of attention and cognitive function postoperatively. A large body of literature has reported that cognitive dysfunction is associated with inflammation (Casserly & Topol, 2004; Granic et al., 2009; van Gool et al., 2010; Yu et al., 2012). Surgery leads to the release of inflammatory factors, and their penetration into the brain through the BBB, which induces neuroinflammation (Mattsson et al., 2011; Rochfort & Cummins, 2015). A study showed that NLR in peripheral blood can also be used as a simple SIR marker and has diagnostic value for certain diseases (Celikbilek et al., 2013). NLR reflects the balance between neutrophils and lymphocytes and is a widely available and easily accessible marker of inflammation (Zha et al., 2021). However, the relationship between NLR and POD has not received much attention. The aim of this study was to investigate the possible relationship among NLR, POD, and CSF biomarkers for the prevention and treatment of clinical POD.

Studies have shown that Aβ_42_, T‐tau, and P‐tau are associated with the development of POD (Buée et al., 2000; Chan et al., 2021; Xie et al., 2014). In addition to causing neuronal degeneration, the activation of a series of pathological events is associated with Aβ deposition. Activation of astrocytes and microglia, dysfunction of the BBB, and microcirculation alterations in microcirculation have been included. All these factors are associated with POD.

Analysis of another study showed that anti‐inflammatory treatment alleviated neuroinflammation and cognitive dysfunction (Grilli et al., 1996). Disruption of the BBB can induce or exacerbate neurodegeneration, leading to cognitive dysfunction (Bell & Zlokovic, 2009). Disruption of the BBB blocks the flow of oxygen and nutrients to the brain, allowing toxins from around the brain to enter the central nervous system (CNS) (Bowman et al., 2009; Kisler et al., 2017). NLR is a marker of endothelial dysfunction (Zha et al., 2021), and accumulation of neutrophils may lead to chronic BBB damage (Abdul Muneer et al., 2011). Neutrophils have been shown to catabolize the BBB (Jickling et al., 2014). Furthermore, if neutrophils infiltrate in the CNS, they exacerbate the cognitive dysfunction. Previous studies have reported increased NLR in elderly patients with delirium (Egberts & FU, 2017). NLR has been shown to be a good predictor of neurological and psychiatric disease outcomes (Hsieh et al., 2012; Kulaksizoglu & Kulaksizoglu, 2016; Xue et al., 2017). This suggests that NLR is closely associated with delirium. These results are similar to our study.

In conclusion, this study suggests that elevated preoperative NLR may be a risk factor for POD and may contribute to POD through elevated P‐tau/T‐tau levels. What is more, Aβ_42_ may be beneficial in reducing the incidence of POD, but the relationship with NLR was not significant. This suggests that preoperative NLR has the potential to serve as a predictor of POD occurrence and further provides a possible mechanism for POD occurrence. In addition, the decision curve and nomogram also suggest that NLR and CSF biomarkers have good predictive effects.

The use of NLR as a target marker in this study has the advantage of being easily available and a common clinical marker of inflammation. The sample size is large and has good clinical reference value. However, this study also has some limitations: First, it is a single‐center trial, and a multicenter study is still needed. Second, my research included different types of surgery, which are probably different risk factors for POD development. Third, this study focused only on the relationship between NLR and Alzheimer‐related biomarkers, and there may be other mechanisms related to inflammation. What is more, larger sample size is needed to further validate.

## 5 CONCLUSION

Elevated preoperative NLR level may be a risk factor for POD and may contribute to POD through elevated P‐tau/T‐tau levels. However, the incidence of POD may be lower in those with higher preoperative Aβ_42_ levels.

Preoperative NLR has the potential to serve as a predictor of POD occurrence. We can reduce the incidence of POD by possible interventions to bring the preoperative NLR level below a certain value. Therefore, this will be the goal of our next study.

## AUTHOR CONTRIBUTIONS

Xiaoyue Wu was the first author of this article, responsible for writing articles and the implementation of this project. Feifei Chi, Bin Wang, Siyu Liu, and Fei Wang were responsible for the data statistics. Jiahan Wang and Xinhui Tang were responsible for the data collection. Yanlin Bi, Xu Lin, and Jun Li modified the manuscript. All authors read and approved the final manuscript.

## CONFLICT OF INTEREST STATEMENT

The authors declare that they have no conflicts of interest.

### PEER REVIEW

The peer review history for this article is available at https://publons.com/publon/10.1002/brb3.3281.

6

**TABLE 1:** Characteristics of participants in perioperative neurocognitive disorder risk factor and prognosis (PNDRFAP) and perioperative neurocognitive disorder and biomarker lifestyle (PNDABLE).

	PNDRFAP			PNDABLE		
Characteristic	POD	Non‐POD	*p*	POD	Non‐POD	*p*
Age [year, M(*Q*)]	73 (65, 81)	65 (58, 71)	.00	74 (71, 78)	61 (54, 67)	.00
Male [*n* (%)]	104 (68)	472 (55.8)	.02	91 (59.1)	495 (48.1)	.36
Education [year, M(*Q*)]	8 (5, 9)	9 (8, 12)	.18	9 (6, 12)	9 (9, 12)	.90
MMSE [scores, M(*Q*)]	25 (24, 26)	27 (26, 28)	.00	28 (27, 29)	28 (27, 30)	.00
Smoking history [*n* (%)]	63 (41.2)	249 (29.4)	.40	38 (24.7)	235 (22.8)	.47
CHD [*n* (%)]	57 (37.3)	170 (20.1)	.39	36 (23.4)	85 (8.3)	.81
Hypertension [*n* (%)]	86 (56.2)	381 (45)	.14	76 (49.4)	284 (27.6)	.14
Diabetes [*n* (%)]	53 (34.6)	173 (20.4)	.22	45 (29.2)	115 (11.2)	.16
NLR [M(*Q*)]	2.79 (1.80, 8.0)	1.85 (1.34, 2.79)	.00	3.21 (1.82, 8.21)	1.73 (1.29, 2.62)	.00
Aβ_40_ [pg/mL, M(*Q*)]	–	–	–	6396.32 (4649.58, 8469.81)	6145 (4327.7, 7998.27)	.28
Aβ_42_ [pg/mL, M(*Q*)]	–	–	–	251.30 (143.54, 420.00)	389.72 (262.08, 558.25)	.02
P‐tau [pg/mL, M(*Q*)]	–	–	–	79.83 (69.33, 97.41)	37.99 (29.67, 46.96)	.00
T‐tau [pg/mL, M(*Q*)]	–	–	–	271.65 (199.08, 565.06)	192.44 (145.92, 260.15)	.00

*Note*: Continuous variables use Student's *t* test or Mann–Whitney *U*, categorical variables use chi‐square test. The numerical variables of normal distribution are statistically described by average ± standard deviation [*x* ± SD]. Non‐normally distributed numerical variables are statistically described by median (interquartile spacing) [M(Q)]. Categorical variables are statistically described by sample size (%) [*n* (%)].

Abbreviations: Aβ_40_, β‐amyloid40; Aβ_42_, β‐amyloid42; MMSE, mini‐mental state examination; NLR, neutrophil‐to‐lymphocyte ratio; POD, postoperative delirium; P‐tau, phosphorylated tau T‐tau, total tau.

**TABLE 2:** Logistic regression on analysis and sensitivity analysis in perioperative neurocognitive disorder risk factor and prognosis (PNDRFAP) study.

	Model 1		Model 2		Model 3		Model 4	
	OR (95% CI)	*p*	OR (95% CI)	*p*	OR (95% CI)	*p*	OR (95% CI)	*p*
NLR	1.158 (1.107–1.212)	.000	1.172 (1.110–1.238)	.000	1.172 (1.108–1.239)	.000	1.067 (1.020–1.116)	.005

*Note*: Model 1: unadjusted; Model 2: adjusted for age (40–90), gender, and MMSE scores; Model 3: Adjusted for age (40–90), gender, MMSE scores, years of education, hypertension, diabetes and coronary heart disease; Model 4: Adjusted for age (≥65), gender, MMSE scores, years of education, hypertension, diabetes and coronary heart disease.

Abbreviations: 95% CI, 95% confidence interval; NLR, neutrophil‐to‐lymphocyte ratio; OR, odds ratio.

**TABLE 3:** Logistic regression on analysis and sensitivity analysis in perioperative neurocognitive disorder and biomarker lifestyle (PNDABLE) study.

	Model 1		Model 2		Model 3		Model 4	
	OR (95% CI)	*p*	OR (95% CI)	*p*	OR (95% CI)	*p*	OR (95% CI)	*p*
NLR	1.208 (1.122–1.301)	.000	1.169 (1.067–1.280)	.001	1.170 (1.064–1.286)	.001	1.182 (1.048–1.335)	.007
Aβ_40_ (pg/mL)	1.000 (1.000–1.000)	.251	1.000 (1.000–1.000)	.252	1.000 (1.000–1.000)	.279	1.000 (1.000–1.000)	.078
Aβ_42_ (pg/mL)	.998 (.996–.999)	.003	.998 (.997–1.000)	.020	.998 (.997–1.000)	.020	.998 (.996–1.000)	.016
P‐tau (pg/mL)	1.099 (1.082–1.116)	.000	1.090 (1.071–1.108)	.000	1.090 (1.071–1.109)	.000	1.083 (1.064–1.102)	.000
T‐tau (pg/mL)	1.003 (1.001–1.004)	.001	1.003 (1.001–1.004)	.003	1.003 (1.001–1.005)	.002	1.003 (1.001–1.005)	.002

*Note*: Model 1: unadjusted; Model 2: adjusted for age (40–90), gender, and MMSE scores; Model 3: adjusted for age (40–90), gender, MMSE scores, years of education, hypertension, diabetes and coronary heart disease; Model 4: adjusted for age (≥65), gender, MMSE scores, years of education, hypertension, diabetes and coronary heart disease.

Abbreviations: 95% CI, 95% confidence interval; NLR, neutrophil‐to‐lymphocyte ratio; OR, odds ratio.

## Data Availability

The raw data supporting the conclusion of this article will be available by the authors, without undue reservation.
